# A Diagnostic Dilemma of Prevertebral Abscess Versus Food Bolus on Lateral Neck X-Ray: A Case Report

**DOI:** 10.7759/cureus.57999

**Published:** 2024-04-10

**Authors:** Alexander Mitropoulos, Stephen Pianko, Ronnie Ptasznik, Jacqueline Fraser

**Affiliations:** 1 Internal Medicine, Monash Medical Centre, Clayton, AUS; 2 Gastroenterology, Monash Health, Melbourne, AUS; 3 Radiology, Monash Health, Melbourne, AUS

**Keywords:** case report, mottled gas, prevertebral abscess, x-ray, neck, food bolus

## Abstract

In this case, a 76-year-old female presenting with globus sensation post-oral intake demonstrated radiographical evidence of mottled radiolucency and prevertebral widening on a lateral neck X-ray at the inferior C4/cricoid cartilage, leading to concern for a prevertebral abscess. A decision was made to proceed with an urgent gastrointestinal endoscopy, and a food bolus was identified and removed, leading to a full remission of the patients’ symptoms. In this case, an appropriate diagnosis was achieved by combining multiple investigations, which highlights to clinicians that taking investigations in isolation, with the aforementioned lateral neck X-ray being the primary example, could lead to potential misdiagnosis and mismanagement of patients.

## Introduction

In the field of medicine, a diagnosis generally incorporates a combination of diagnostic tools, including clinical presentation, pathological testing, and radiological imaging. The following case provides a reminder to all medical professionals by demonstrating how such diagnostic tools taken in isolation may potentially lead to a misdiagnosis and that there is a recognized need to consider them collectively [1]. The case is unique as it reflects real-time decision-making in a hospital setting, highlights the dangers of not combining investigations, and simultaneously showcases the fast progression of a food bolus to manifest radiologically as a prevertebral abscess.

## Case presentation

A 76-year-old female was brought in by ambulance from a high-level care nursing home with globus sensation approximately 2 hours post-oral intake of lamb. This was accompanied by vomiting, chest pain, and an inability to tolerate oral secretions. Relevant past medical history included Alzheimer’s dementia and mild oropharyngeal dysphagia.

**Figure 1:**
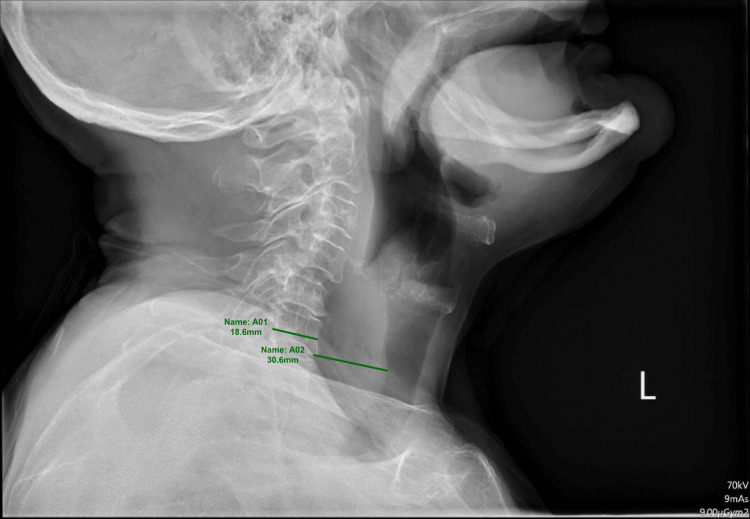
X-ray soft tissue neck A01: C6 mid-vertebral anteroposterior diameter (18.6mm) A02: C6 pre-vertebral soft tissue space widening (30.6mm)

A routine soft tissue radiograph of the lateral neck was performed within 6 hours of presentation, as shown in Figure 1. A formal report was pending before the endoscopy. The soft tissue X-ray of the neck demonstrated prevertebral thickening of 30.6mm (3.06cm), accompanied by the appearance of mottled gas. The patient underwent an upper-gastrointestinal endoscopy. Endoscopy revealed an impacted food bolus in the proximal esophagus with concurrent inflammation and edema at 15cm from the incisors. The food bolus was successfully removed with rat-toothed forceps. The procedure was uncomplicated, and the patient discharged home after tolerating oral intake. 

## Discussion

The radiologist’s final report of the soft tissue lateral radiograph of the neck revealed significantly thickened (3.06 cm) prevertebral soft tissues inferior to the C4/cricoid cartilage at approximately 17cm from the incisors [2]. This widening showed subtle mottled radiolucency within it, thus raising the differential of a prevertebral abscess [3,4]. However, the temporal relationship with the food bolus, normal white cell count, lack of fever, and full cervical range of motion weighed in favor of the food bolus as the causative agent [4,5]. A decision was made by the gastroenterologist to proceed with endoscopy, in which the food bolus was removed, and the patient had subsequent resolution of symptoms. This demonstrates the necessity of using a combination of investigations in order to obtain an appropriate diagnosis. If the radiograph results were taken in isolation, this patient could potentially have been misdiagnosed and mistreated as a prevertebral abscess, thus delaying the causative agent of a food bolus from being removed. In the circumstance that this was a prevertebral abscess, endoscopy would still have proved beneficial as it is a relatively expeditious procedure, would eliminate the differential of a food bolus, and would help to further characterize the prevertebral abscess for intervention. Although not performed, further imaging, such as a CT neck, could have been considered; however, the gastroenterologist's opinion was that this was a food bolus, and in the interest of time, endoscopy was rapidly undertaken. It is important to recognize that when patients present with life-threatening symptoms in the clinical setting, decisions are being made promptly and with expert clinical judgment. Another area of particular interest in this case was the ability of the food bolus to radiologically manifest in a manner that can mimic a prevertebral abscess, in conjunction with the short time interval of 8 hours from oral intake, to produce such significant radiological findings. The reason for this is not entirely clear and should remind clinicians that there will be instances in which suspicious or unexpected findings will be encountered. It would prove interesting in the future to compare this case to other cases of food bolus to determine whether they reflect a similar time pattern between the occurrence of the food bolus and the development of radiographic findings.

## Conclusions

This case serves as a reminder for clinicians to be prepared for unexpected findings and how investigations in isolation may promote more than one differential diagnosis. In this case study, if radiological imaging was considered alone, the patient may have been misdiagnosed and mismanaged as a prevertebral abscess rather than a food bolus. This was overcome by correlating several investigative tools, including radiological imaging, pathological investigations, and clinical presentation, which resulted in endoscopic removal of the food bolus and a resolution of patient symptoms. All clinicians should thereby aim to use a multitude of investigative measures, as this will likely lead to more accurate diagnoses and appropriate intervention for patients. 
